# mRNA and protein dataset of autophagy markers (LC3 and p62) in several cell lines

**DOI:** 10.1016/j.dib.2016.02.085

**Published:** 2016-03-09

**Authors:** Rubén Gómez-Sánchez, Sokhna M.S. Yakhine-Diop, Mario Rodríguez-Arribas, José M. Bravo-San Pedro, Guadalupe Martínez-Chacón, Elisabet Uribe-Carretero, Diana C.J. Pinheiro de Castro, Elisa Pizarro-Estrella, José M. Fuentes, Rosa A. González-Polo

**Affiliations:** a Centro de Investigación Biomédica en Red en Enfermedades Neurodegenerativas (CIBERNED), Spain; b Department of Cell Biology, University of Groningen, University Medical Center Groningen, A. Deusinglaan 1, 9713 AV Groningen, The Netherlands; c Departamento de Bioquímica, Biología Molecular y Genética. Facultad de Enfermería y Terapia Ocupacional. Avda de la Universidad S/N C.P, 10003 Cáceres, Spain; d Equipe 11 labellisée Ligue contre le Cancer, Centre de Recherche des Cordeliers, 75006 Paris, France; e INSERM U1138, 75006 Paris, France; f Université Paris Descartes/Paris V, Sorbonne Paris Cité, 75006 Paris, France; g Université Pierre et Marie Curie/Paris VI, 75006 Paris, France; h Gustave Roussy Comprehensive Cancer Institute, 94805 Villejuif, France

**Keywords:** Baf. A1, Bafilomycin A1, EBSS, Earle׳s Balanced Salt Solution, FBS, Fetal bovine serum, GAPDH, Glyceraldehyde 3-phosphate dehydrogenase, HFs, Human fibroblasts, LC3, Microtubule-associated protein 1 light chain 3, MEFs, Mouse embryonic fibroblasts, PBS, Phosphate-buffered saline, qPCR, Quantitative PCR, SB, Sample buffer, SDS, Sodium dodecyl sulfate, TBST, Tris-buffered saline with Tween 20, Autophagy, LC3, p62, Western-blot

## Abstract

We characterized the dynamics of autophagy *in vitro* using four different cell systems and analyzing markers widely used in this field, i.e. LC3 (microtubule-associated protein 1 light chain 3; protein recruited from the cytosol (LC3-I) to the autophagosomal membrane where it is lipidated (LC3-II)) and p62/SQSTM1 (adaptor protein that serves as a link between LC3 and ubiquitinated substrates), (Klionsky et al., 2016) [1]. Data provided include analyses of protein levels of LC3 and p62 by Western-blotting and endogenous immunofluorescence experiments, but also *p62* mRNA levels obtained by quantitative PCR (qPCR). To monitor the turnover of these autophagy markers and, thus, measure the flux of this pathway, cells were under starvation conditions and/or treated with bafilomycin A1 (Baf. A1) to block fusion of autophagosomes with lysosomes.

**Specifications Table**

**Table:** 

Subject area	*Biochemistry and Molecular Biology*
More specific subject area	*Autophagy*
Type of data	*Figures*
How data was acquired	*Western-blotting (SDS-gel electrophoresis and semi-dry transfer; Bio-Rad equipment and ImageJ software), immunofluorescence (inverted microscope (OLYMPUS IX-51) and ImageJ software), quantitative PCR (qPCR) (Applied Biosystems 7500 PCR real system), data statistical analysis (SPSS software).*
Data format	*Analyzed*
Experimental factors	*Four cell lines (Mouse embryonic fibroblasts (MEFs), Human fibroblasts (HFs), SH-SY5Y human neuroblastoma cells, and N27 rat dopaminergic cells). Starvation-induced autophagy by incubation with Earle׳s Balanced Salt Solution (EBSS) medium, bafilomycin A1 (Baf. A1) treatment (blocking autophagosome-lysosome fusion), and dual treatment (Baf. A1+EBSS).*
Experimental features	*Western-blotting analysis of LC3 and p62 proteins using Sample buffer (SB) 1X lysis buffer, detection of endogenous LC3 and p62 immunofluorescence and p62 mRNA levels by qPCR.*
Data source location	*Not applicable*
Data accessibility	*Data is within this article*

**Value of the data**

• This data provides characterization of basal macroautophagy flux and response to classical inducers (EBSS) and inhibitors (Baf. A1) of this mechanism in MEFs, HFs, SH-SY5Y and N27 cell lines.
• The data would be valuable for further studies of autophagy dynamics in these four cell lines.
• This data could give a basis for further experiments on revealing the underlying mechanism of autophagy in these cell lines.
• The data support the development of transcription analysis of autophagy markers.

## 1. Data

**Fig. 1:**
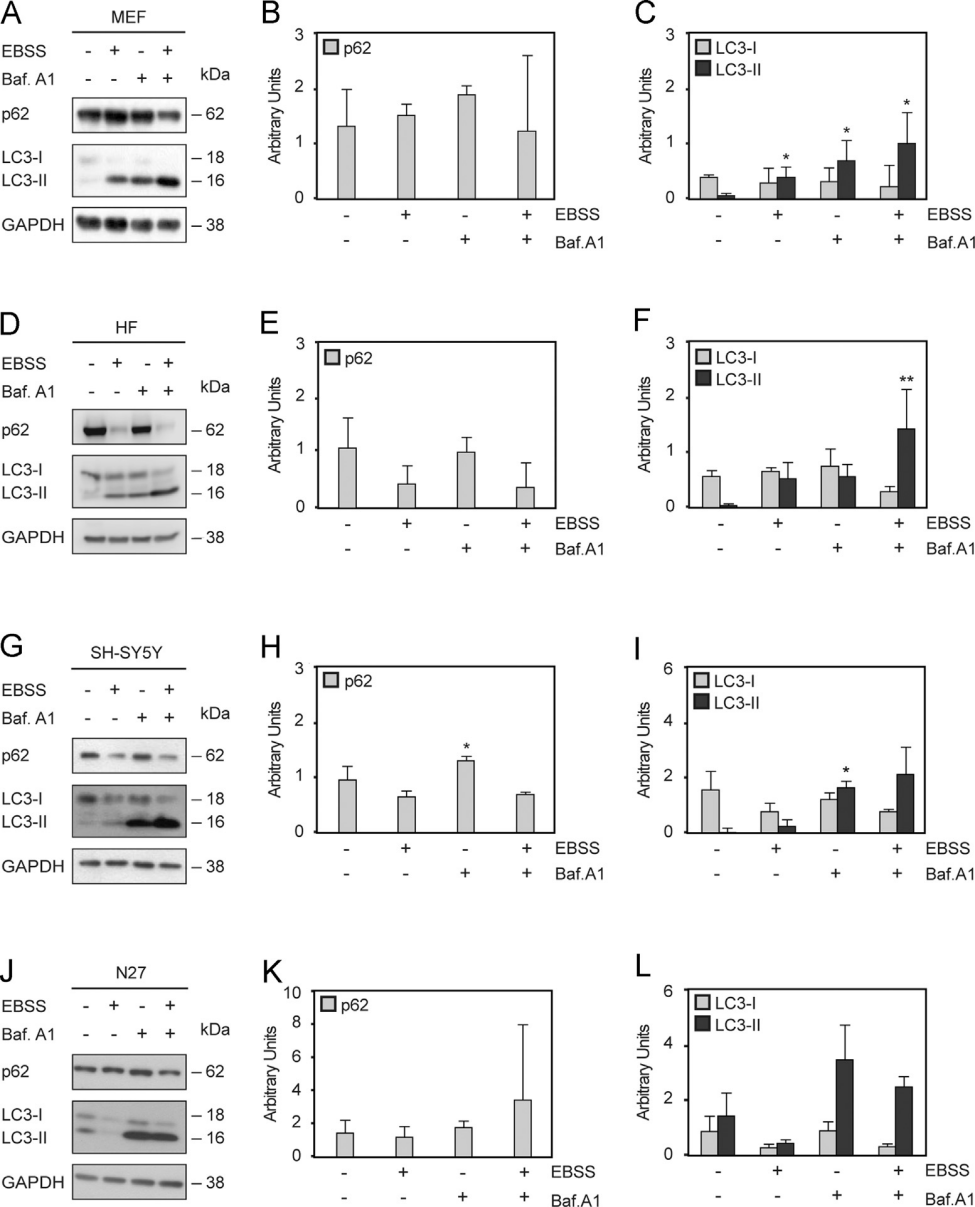
*LC3 and p62 modulation after EBSS±Baf. A1 treatment in four cell models.* Cells were treated with EBSS, Baf. A1 and dual treatment for 4 h, as described in Section 2. Representative blots of 3 independent experiments of MEFs (A), HFs (D) SH-SY5Y (G) and N27 rat dopaminergic cells (J) are shown. GAPDH was used as a loading control. p62 densitometry expressed in arbitrary units is shown in panels B (MEFs), E (HFs), H (SH-SY5Y) and K (N27). LC3 (isoforms I and II) densitometry expressed in arbitrary units is shown in panels C (MEFs), F (HFs), I (SH-SY5Y) and L (N27). Data represent the mean±SEM; *n*=3 (*p≤0.05, **p≤0.01, ***p≤0.001, related to the corresponding non-treated condition).

**Fig. 2:**
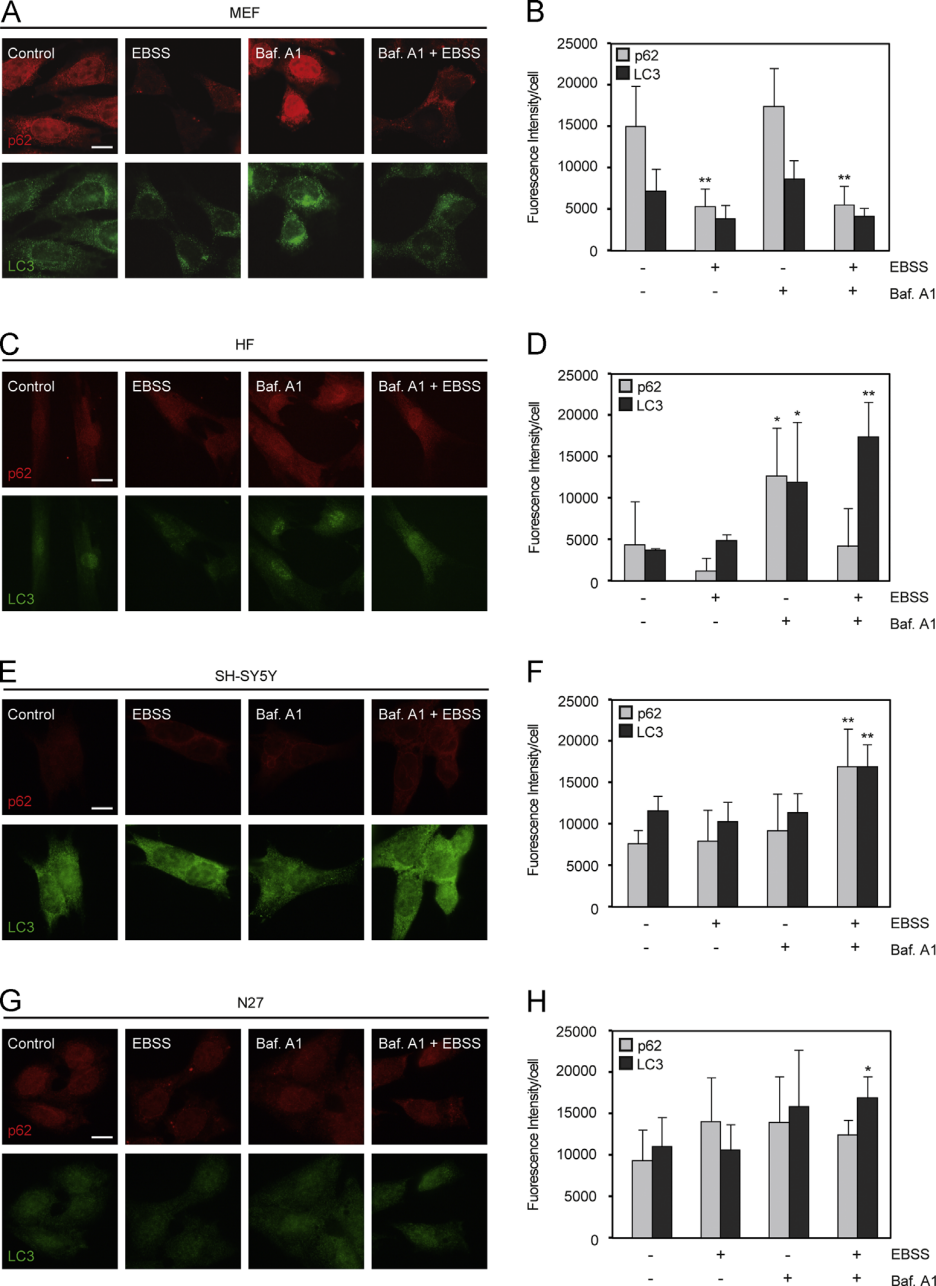
*Endogenous immunofluorescence of LC3 and p62 after EBSS ± Baf. A1 treatment in four cell models*. Cells were treated with EBSS, Baf. A1 and dual treatment for 4 h, as described in Section 2. Representative images from MEFs (A), HFs (C), SH-SY5Y (E) and N27 cells (G) are shown. Scale bars equate to 10 µm. Histograms show quantification of fluorescence intensity per cell from MEFs (B), HFs (D), SH-SY5Y (F) and N27 cells (H). Data represent the mean±SEM; *n*=3 (*p≤0.05, **p≤0.01, related to the corresponding non-treated condition).

**Fig. 3:**
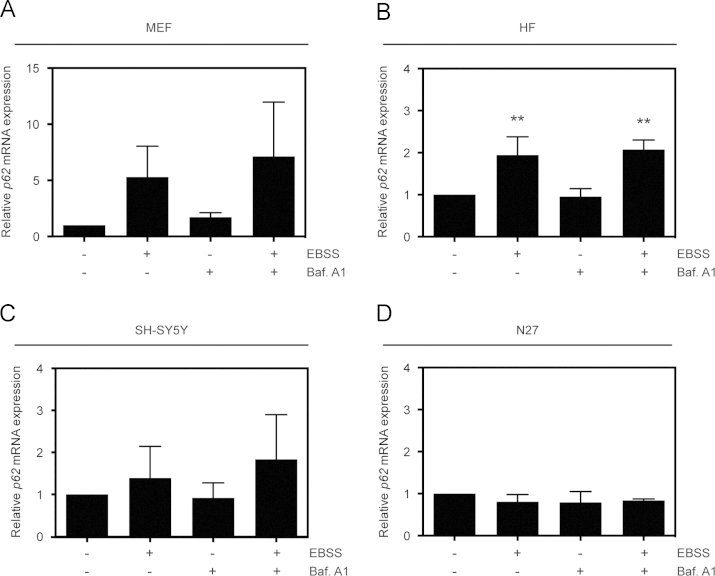
*p62 mRNA analysis after EBSS±Baf. A1 treatment in four cell models.* Cells were treated with EBSS, Baf. A1 and dual treatment for 4 h, as described below. Quantification of *p62* mRNA expression levels from all lines studied is shown: MEFs (A), HFs (B), SH-SY5Y (C) and N27 cells (D). Relative expression was determined using *GAPDH* as a housekeeping gene. Data represent the mean±SEM; *n*=3 (**p≤0.01, related to the corresponding non-treated condition).

In order to understand autophagy dynamics [1] and develop a robust methodological assay, we used four cell models: MEFs, HFs, SH-SY5Y human neuroblastoma cells, and N27 rat mesencephalic dopaminergic cells. LC3 and p62 protein levels were analyzed by Western-blotting assay, using SB 1X as a lysis buffer (Fig. 1). Furthermore, we measured the endogenous immunostaining of LC3 and p62 proteins in all cell models used in this report (Fig. 2). To complete this data analysis, *p62* mRNA expression was performed in all cell lines (Fig. 3).

## 2. Experimental design, materials and methods

### 2.1. Cell culture and treatments

To perform this data analysis, we used four cell lines: MEFs, HFs, SH-SY5Y and N27 rat mesencephalic dopaminergic cells. The culture media for MEFs, HFs and SH-SY5Y were Dulbecco׳s Modified Eagle Medium-High Glucose (Sigma-Aldrich, D6546) supplemented with 10% fetal bovine serum (FBS) (Sigma-Aldrich, F7524), 1% L-glutamine (Sigma-Aldrich, G7513) and penicillin-streptomycin (Hyclone, SV30010). For N27 cell line, the culture medium was made of RPMI 1640 medium (1X) (Hyclone, SH30096.01) supplemented with 10% FBS, L-glutamine and penicillin-streptomycin.

Cells were seeded at densities of 3×10^5^ (MEFs), 1×10^6^ (HFs), 2×10^6^ (SH-SY5Y) and 4×10^5^ (N27) in 75-cm^2^ tissue culture flasks and incubated at 37 °C under saturating humidity in 5% CO_2_/95% air. Confluent cells (~80%) were trypsinized and plated into a 6 or 24-well plates at densities of 3×10^4^ cells/mL (MEFs and HFs), 1×10^5^ cells/mL (SH-SY5Y) and 3.5×10^4^ cells/mL (N27).

After 24 h , the culture medium was replaced with different treatments (Control, EBSS, Baf. A1 and Baf. A1+EBSS). To induce autophagy by starvation conditions, the culture medium was replaced with EBSS (Sigma-Aldrich, E2888). To block fusion between autophagosomes and lysosomes, cells were incubated with 100 nM Baf. A1 (LC Laboratories, B-1080). For combined treatment, cells were preincubated with 100 nM Baf. A1 for 1 h and then they were washed with phosphate-buffered saline (PBS) 1X and treated with 100 nM Baf. A1 and EBSS. All treatments lasted 4 h.

### 2.2. Western-blotting

Following treatments, cells were processed as described previously [2]. Basically, cells were washed with PBS 1X and lysed in sample buffer (SB) 1X (2% (v/v) sodium dodecyl sulfate (SDS), 10% (v/v) glycerol, and 50 mM Tris–HCl, pH 6.8, in distilled water) by pipetting until homogenization. Protein concentration was measured based on the bicinchoninic acid assay, using bovine serum albumin as a standard. Samples were heated at 95 °C for 10 min before their quantification.

Equal amounts of protein (25–40 µg/condition) were resolved by 12% SDS-gel electrophoresis and transferred to polyvinylidene fluoride membranes, according to a partially modified conventional protocol [3]. Immunodetection included transferring (15 V during 15 min, per each membrane) and blocking of the membrane with WB blocking solution (10% w/v fat free milk in Tris-buffered saline with Tween 20 (TBST)) for 1 h at room temperature. After washing the membranes 2 times with TBST 1X, blots were incubated with the corresponding primary antibody: p62/SQSTM1 (1:5000) (BD Transduction Laboratories, 610498), LC3-B (1:5000) (Sigma–Aldrich, L7543) and glyceraldehyde 3-phosphate dehydrogenase (GAPDH) (1:5000) (Millipore, MAB374) at 4 °C overnight, GAPDH (1 h at room temperature). The membranes were washed 2 times with TBST 1X and subsequently incubated with their respective HRP-conjugated secondary antibodies (1:10000) (Bio-Rad, 170–6515 and 170-5047 for rabbit and mouse antibodies, respectively) for 1 h at room temperature. Detection of bound antibodies was visualized by chemiluminescence using ECL substrate (Thermo Scientific, 32106). Quantification data analysis was performed using ImageJ software (NIH), establishing GAPDH protein levels as a loading control.

### 2.3. Immunofluorescence

For the detection of endogenous p62 and LC3B, cells were seeded on coverslips, fixed with paraformaldehyde (4% in PBS 1X) and permeabilized with Triton X-100 solution (0.1% in PBS 1X) for 10 min. To block non-specific binding, cells were incubated with 10% FBS in PBS 1X for 20 min, followed by incubation with primary antibodies anti-p62 (1:500) and anti-LC3B (1:500) for 1 h at room temperature. After that, cells were incubated with Alexa Fluor 488 anti-rabbit (1:1000) (Molecular Probes, A-11034) and 568 anti-mouse (1:1000) (Molecular Probes, A-11004) secondary antibodies for LC3 and p62, respectively. Finally, coverslips were mounted on microscope slides, by using fluoromount-G (SouthernBiotech, 0100–01) medium. Images were taken by using an inverted fluorescence microscope (Olympus, IX-51) equipped with a camera (Olympus, DP70). The quantitative measurement of the fluorescence signal was performed using ImageJ software analyzing at least 200 cells per condition. Immunofluorescence procedure was developed as previously described [2].

### 2.4. Quantitative PCR

Total RNA was extracted by RNeasy Mini Kit (Qiagen, 74104); 500 ng of total RNA were reverse-transcribed into complementary DNA by using a QuantiTect Reverse Transcription Kit (Qiagen, 205311), both according to the manufacturer׳s protocol. *p62* mRNA expression was measured by qPCR with KAPA SYBR Fast reagents (KK4601), by using the primers described above. *GAPDH* gene expression was used as an endogenous control, and the expression level was calculated by using the (2^-∆∆Ct^) ratio [4].

### 2.5. Statistical analyses

Each experiment was repeated at least three times. The data were analyzed by two-tailed unpaired Student׳s t-test and ANOVA test where applicable, and all comparisons with a p value less than 0.05 (*p*<0.05) were considered statistically significant (***p<0.001, **p<0.01, *p<0.05). Non-significant results are not indicated in the figures. The data are expressed as the mean±the standard error of the mean (SEM).
